# 

**Published:** 1885-06

**Authors:** 


### THE BRISTOL MEDICO-CHIRURGICAL JOURNAL

A Quarterly Journal of the Medical Sciences for the West of England and South Wales

PUBLISHED UNDER THE AUSPICES OF

THE BRISTOL MEDICO-CHIRURGICAL SOCIETY.

EDITED BY

J. GREIG SMITH,

M.A., F.R.S.E.

"Scire est nescire, nisi id me

Scire alius sciret."

BRISTOL: J. W. ARROWSMITH;
LONDON: J. & A. CHURCHILL, 11 NEW BURLINGTON STREET.

*Price One Shilling and Sixpence.*

